# Rationale and Design of the Genotype-Blinded Trial of Torasemide for the Treatment of Hypertension (BHF UMOD)

**DOI:** 10.1093/ajh/hpaa166

**Published:** 2020-10-21

**Authors:** Linsay McCallum, Katriona Brooksbank, Alex McConnachie, Alisha Aman, Stefanie Lip, Jesse Dawson, Iain M MacIntyre, Thomas M MacDonald, David J Webb, Sandosh Padmanabhan

**Affiliations:** 1 Institute of Cardiovascular and Medical Sciences, BHF Glasgow Cardiovascular Research Centre, University of Glasgow, Glasgow, UK; 2 Robertson Centre for Biostatistics, Institute of Health and Wellbeing, University of Glasgow, Glasgow, UK; 3 Clinical Pharmacology Unit and Research Centre, University of Edinburgh/BHF Centre of Research Excellence, Edinburgh, UK; 4 MEMO Research, University of Dundee, Ninewells Hospital and Medical School, Dundee, UK

**Keywords:** blood pressure, furosemide, hypertension, loop-diuretic, NKCC2, uromodulin

## Abstract

**BACKGROUND:**

Genome-wide association studies have identified single nucleotide polymorphisms (SNPs) near the uromodulin gene (*UMOD*) affecting uromodulin excretion and blood pressure (BP). Uromodulin is almost exclusively expressed in the thick ascending limb (TAL) of the loop of Henle and its effect on BP appears to be mediated via the TAL sodium transporter, NKCC2. Loop-diuretics block NKCC2 but are not commonly used in hypertension management. Volume overload is one of the primary drivers for uncontrolled hypertension, so targeting loop-diuretics to individuals who are more likely to respond to this drug class, using the *UMOD* genotype, could be an efficient precision medicine strategy.

**METHODS:**

The BHF UMOD Trial is a genotype-blinded, multicenter trial comparing BP response to torasemide between individuals possessing the AA genotype of the SNP rs13333226 and those possessing the G allele. 240 participants (≥18 years) with uncontrolled BP, on ≥1 antihypertensive agent for ≥3 months, will receive treatment with Torasemide, 5 mg daily for 16 weeks. Uncontrolled BP is average home systolic BP (SBP) >135 mmHg and/or diastolic BP >85 mmHg. The primary outcome is the change in 24-hour ambulatory SBP area under the curve between baseline and end of treatment. Sample size was calculated to detect a 4 mmHg difference between groups at 90% power. Approval by West of Scotland Research Ethics Committee 5 (16/WS/0160).

**RESULTS:**

The study should conclude August 2021.

**CONCLUSIONS:**

If our hypothesis is confirmed, a genotype-based treatment strategy for loop diuretics would help reduce the burden of uncontrolled hypertension.

**CLINICAL TRIALS REGISTRATION:**

https://clinicaltrials.gov/ct2/show/NCT03354897.

## Graphical Abstract

**Graphical Abstract:**
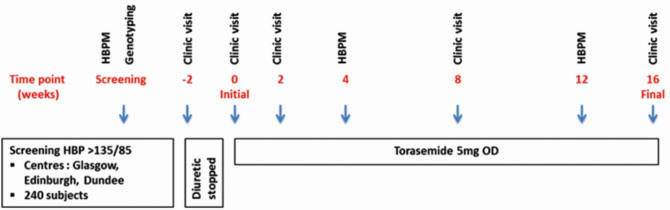

Pharmacotherapy is the mainstay of hypertension management though the selection of agents is essentially by trial-and-error. Six drug classes are commonly used however response rates to any given drug, is around 50%.^1^ Antihypertensive drug development has stalled and there has been little progress in routine stratification by leveraging genomic and molecular information, despite potential benefit.^2,3^ Evidence is emerging of a novel pathway, mediated by the uromodulin (*UMOD*) gene (which is exclusively expressed in the kidney’s thick ascending limb (TAL) of the loop of Henle), that influences blood pressure (BP), with the potential to improve hypertension management and stimulate drug discovery.^4^ In a large genome wide association study of BP extremes, the minor G allele of a *UMOD* promoter single-nucleotide polymorphism (SNP), rs13333226, was associated with a lower risk of hypertension (odds ratio [95% confidence interval]: 0.87 [0.84; 0.91]) and reduced urinary uromodulin excretion.^5^ There are 3 SNPs in complete linkage disequilibrium in the UMOD promoter region, rs12917707(G>T), rs4293393(A>G), and rs13333226(A>G) which show similar associations in multiple genome wide association study studies. Each copy of the minor allele of these SNPs is associated with a lower risk of hypertension (odds ratio [95% confidence interval]: 0.87 [0.84–0.91], *P* = 3.6 × 10^−11^),^5^ 0.49 mm Hg lower systolic BP (SBP) and 0.30 mm Hg lower diastolic BP (DBP) (*P* < 3 × 10^−5^),^5,6^ higher estimated glomerular filtration rate (−0.0158 (SE = 0.0011), *P* = 9.47 × 10^−43^),^7^ decreased risk of chronic kidney disease (1.24 [1.19–1.29], *P* = 1.98 × 10^−25^),^7^ and a lower level of urinary uromodulin excretion (−0.31 (0.018), *P* = 3.4 × 10^−64^).^8^ Homozygous carriers of the risk allele (rs13333226(A), rs12917707(G), and rs4293393(A)) have twofold higher levels of uromodulin in the urine, compared with homozygous carriers of the minor (rs13333226(G), rs12917707(T), and rs4293393(G)) protective allele.^9^ Mechanistic studies in mouse models showed *UMOD* knockout mice had significantly lower SBP than wild-type mice, were resistant to salt-induced BP changes and demonstrated a left shift of the pressure–natriuresis curve.^10^ Trudu *et al.* showed that *UMOD* overexpression caused a dose dependent increase in *UMOD* expression, associated with a BP increase.^9^ They also showed that loop-diuretic treatment significantly enhanced natriuresis and reduced BP levels both in the transgenic mouse and in hypertensive individuals homozygous for the *UMOD* increasing allele. These findings point to a novel pathway of BP and renal function regulation through interaction between the key Na^+^–K^+^–2Cl^−^ cotransporter (NKCC2) in the TAL, which is specifically blocked by loop-diuretics, and uromodulin. These studies raise the possibility that hypertensive patients who possess the *UMOD* increasing allele will respond better to loop-diuretics. As volume overload is considered one of the primary drivers for uncontrolled hypertension, targeting loop-diuretics to individuals who are more likely to respond to this class would be a precision medicine strategy to improve population hypertension control.

## STUDY HYPOTHESIS

Our hypothesis is that based on the *UMOD* rs13333226 genotype, there exists 2 strata of hypertensive patients who will show differential response to loop-diuretics based on their *UMOD* genotype. The high-UMOD group (homozygous for the major allele, AA genotype) with increased uromodulin excretion, greater salt sensitivity, hypertension, normal estimated glomerular filtration rate, and greater BP response to the loop-diuretic torasemide and the low-UMOD group (1 or 2 copies of the G allele) with decreased uromodulin excretion, greater salt resistance, increased estimated glomerular filtration rate, increased proximal tubular reabsorption of Na^+^ (possibly related to increased GFR), a poor BP response to loop-diuretics, and possibly diminished function of NKCC2. Our primary objective is to test whether hypertensive subjects with uncontrolled BP possessing the rs13333226 AA genotype will be better responders to loop-diuretics than those possessing the G allele.

## METHODS

### Study design

The British Heart Foundation (BHF) UMOD trial is a genotype-blinded, multicenter trial which will compare the BP response to torasemide between individuals possessing the AA genotype of the SNP rs13333226 and those possessing the G allele. Funding is by the BHF; CS/16/1/31878.

### Study participants

**Figure 1.**
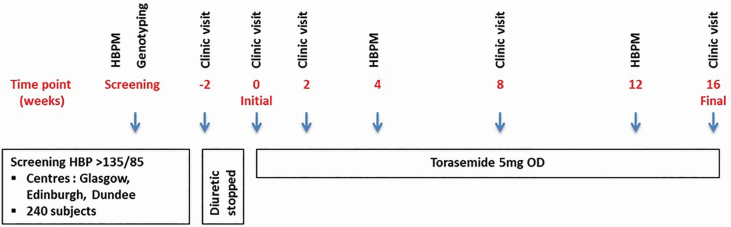
BHF UMOD study flow chart. Abbreviations: HBP, home blood pressure; ECG, electrocardiogram; HBPM, home blood pressure monitoring.

Two hundred and forty participants aged ≥18 years from 3 study sites (Dundee, Edinburgh, and Glasgow) will be prescribed torasemide for 16 weeks. Participants will have uncontrolled BP on ≥1 nondiuretic antihypertensive agent for ≥3 months prior to enrollment. Uncontrolled BP is defined as average SBP >135 mm Hg and/or DBP >85 mm Hg on home BP monitoring (HBPM). Participants already taking a diuretic may be included if it safe to wash out this drug for a 2-week period prior to baseline visit. Study visits will be at week −2 (preexisting diuretics), 0, 2, 8, and 16. The study flow chart is shown in Figure 1 and the detailed participant schedule is shown in Table 1. Full inclusion and exclusion criteria are shown in Table 2.

**Table 1.** Detailed participant schedule

		Treatment phase						
Activity	Screening	Week −2^a^	Week 0	Week 2	Week 4	Week 8	Week 12	Week 16
Clinic visit		X	X	X		X		X
Review eligibility	X	X	X					
Informed consent	X	X	X					
Salivary DNA sample	X							
Demography	X	X	X					
Medical history		X	X					X
Medical examination		X^c^	X^c^					X^c^
Weight, height, BMI		X	X			X		X
Medication review		X	X	X		X		X
Clinic blood pressure		X	X	X		X		X
Blood sampling		X	X	X		X		X
ECG			X^c^					X^c^
Urine sampling			X^b^			X		X^b^
ABPM			X			X		X
HBPM	X				X		X	
Compliance check				X		X		X
Adverse events reporting				X		X		X
Dispense study medication			X			X		

Abbreviations: ABPM, ambulatory blood pressure monitoring; BMI, body mass index; ECG, electrocardiogram; HBPM, home blood pressure monitoring.

^a^If on diuretics prior to study.

^b^Including pregnancy testing for women of childbearing potential.

^c^Not required for all participants, at discretion of the investigator.

**Table 2.** Eligibility criteria

Inclusion criteria
Hypertensive patients aged ≥18 years of age
Patients will all have hypertension that is not controlled to home target: SBP >135 mm Hg and/or DBP >85 mm Hg on therapy with 1 or more antihypertensive drugs for at least 3 months
Able to attend one of the 3 study centers
Exclusion criteria
Inability to give informed consent
Participation in a clinical study involving an investigational drug or device within 3 months of screening
Secondary or accelerated hypertension
Diabetes mellitus (type 1 or type 2)
eGFR <60 ml/minute, hyponatremia, hypokalemia
Pregnancy, breast feeding
Women of childbearing potential who are unwilling to use effective contraception
Childbearing potential is defined as women who have experienced menarche and who have not undergone successful surgical sterilization or who are not postmenopausal (irregular menstrual periods, or amenorrhea >12 months, with serum follicle-stimulating hormone (FSH) >35 mIU/ml; women taking hormone replacement therapy (HRT)
Women of childbearing potential will be eligible if they are willing to use acceptable contraception (combined oral contraceptives, progesterone only contraceptives, intrauterine device, barrier methods) or they are abstinence due to lifestyle choice or their partner is sterile (vasectomy)
Anticipated change of medical status during the trial (e.g., surgical intervention requiring >2 weeks convalescence)
Recent (<6 months) cardiovascular event requiring hospitalization (e.g., myocardial infarction or stroke)
Requirement for study drug or other loop-diuretic for reason other than to treat hypertension
Clinically relevant contraindication to treatment with torasemide: hypersensitivity, hereditary problems of glucose intolerance, Lapp lactase deficiency of glucose–galactose malabsorption
Current therapy for cancer
Concurrent chronic illness, or other reasons likely to preclude 18-week participation in the study
Any concomitant condition that, in the opinion of the investigator, may adversely affect the safety and/or efficacy of the study drug or severely limit that patients life-span or ability to complete the study (e.g., alcohol or drug abuse, disabling or terminal illness, severe liver impairment, mental disorders)
Treatment with any of the following medications
Oral corticosteroids within 3 months of screening. Treatment with systemic corticosteroids is also prohibited during study participation
Chronic stable use, or unstable use of NSAIDs (other than low dose aspirin or occasional over the counter analgesic doses) is prohibited. Chronic use is defined as >3 consecutive days of treatment per week. In addition, intermittent use of NSAIDs is discouraged throughout the study. For those requiring analgesics during the study, paracetamol or opiate drugs are recommended
Use of lithium
Participants on the following medications may be included provided they meet the following criteria
Use of thiazide or loop-diuretics prior to the study if the diuretic can be stopped for 2 weeks (washout) before the study medication administered
The use of short acting nitrates (e.g., sublingual nitroglycerin) is permitted. However, participants should avoid short acting oral nitrates within 4 hours of screening or a subsequent visit
The use of long acting nitrates (e.g., Isordil) is permitted but the dose must be stable for at least 2 weeks prior to screening
The use of sympathomimetic decongestants is permitted, though not within 24 hours of any study visit/BP assessment
The use of theophylline is permitted but the dose must be stable for at least 4 weeks prior to screening and throughout the study
The use of phosphodiesterase type V inhibitors is permitted. However, study participants must refrain from taking these medications for at least 7 days prior to screening or any subsequent study visit

Abbreviations: BP, blood pressure; DBP, diastolic blood pressure; eGFR, estimated glomerular filtration rate; NSAIDs, non-steroidal anti- inflammatory drugs; SBP, systolic blood pressure.

### Recruitment

Potential participants will be identified from secondary care BP clinics and general practice sites.

### Participant consent and withdrawal

Interested participants will visit the study website (www.bhfumod.co.uk) to complete initial consent, confirming they have read the participant information sheet and checking eligibility criteria. A final check box marked “I have read, answered and understood all of the above questions and understand this is an electronic signature” must be ticked before proceeding to enrollment. A copy of the consent form and participant information sheet will be sent to the participant electronically and a copy retained by the researchers. A paper version of the participant information sheet and consent form will be available. Further written informed consent will be given at the initial study visit.

### Screening

Consented, eligible participants will perform HBPM as detailed below. If HBPM criteria are met, a salivary DNA kit is used to collect the DNA sample which is returned to the University of Glasgow laboratory for genotyping. The genotype is entered in the electronic case report form (eCRF) by the laboratory technician who does not have access to any of the trial data. The genotype is not disclosed to any of the trial personnel or the participant.

### Study drug

Torasemide was chosen for this study as it is licensed for use in hypertension^11^ and has a longer half-life than the more commonly prescribed furosemide (4 vs. 1.5 hours), mitigating the secondary Renin Angiotensin System activation, and has a longer duration of action than furosemide (12 vs. 6 hours). This has enabled us to conduct this innovative trial with torasemide without additional regulatory oversight and costs.

Torasemide will be initiated at 5 mg once daily, reducing to 2.5 mg once daily if required. The study drug will be supplied at weeks 0 and 8.

As with other diuretics, there may be disturbances of water and electrolyte balance. Hypokalemia and/or hyponatremia may occur. Other undesirable effects include elevated uric acid level, glucose, lipids, and decreases in platelets, white or red blood cells. There are known interactions with aminoglycosides, cephalosporins, lithium, theophylline, non-steroidal anti-inflammatory drugs, digoxin, and sulphonylureas.

### Drug adherence

Participants will be encouraged to return all unused/empty study medication packets at weeks 2, 8, and 16 for an adherence check (tablet count). Any issues with adherence will be recorded in the eCRF. Participants who are nonadherent will be encouraged and can be reminded by telephone, text, or email.

### Investigator withdrawal

Participants may be withdrawn from the study medication on safety grounds, at investigator discretion. All study drug withdrawals will be recorded in the eCRF. Reasons for investigator withdrawal are found in Table 3. Participants may remain in the study, at investigator discretion, without taking the medication and will not be replaced.

**Table 3.** Clinical reasons for withdrawal of participants

Illness requiring hospitalization that is likely to preclude further study participation.
Known side-effects of the trial drug which are considered intolerable by the participant.
Extreme electrolyte disturbances or renal dysfunction.
Hypotension—If the patient has symptoms consistent with hypotension and clinic systolic BP is <110 mm Hg or the change in BP from previous reading is >30 mm Hg, and in the opinion of the investigator there is no self-limiting reason for hypotension (e.g., episode of dehydration, climatic conditions).

Abbreviation: BP, blood pressure.

### Treatment interruptions

Treatment may be interrupted at investigator discretion. Participant will be reviewed by the investigator who will determine if suitable to continue in the study and when/if the drug is to be restarted. Interruptions will be logged in the eCRF. If an interruption is more than 1 and up to 21 days, the study period may be lengthened. An interruption of more than 21 days will prompt withdrawal from study. Interim study visits may be scheduled as required.

### Adverse events

Adverse events must be assessed and recorded in the participant’s case notes. Only Related Unexpected Serious Adverse Events (RUSAEs) are to be collected in an expedited manner and reported to the sponsor. An RUSAE is an unexpected event that is thought to be related to a trial-specific procedure and/or the study drug, with reference to the most recent SmPC.^11^

RUSAEs will be reported via the eCRF to the Pharmacovigilance Office within 24 hours of the site becoming aware of the event. The local principal investigator (or designee) must ensure that any missing information is entered into the eCRF.

### Post-trial care

At the final study visit, the participant will be reviewed by an investigator who will advise on further antihypertensive therapy and communicate with the participant’s general practice. There is no provision for the supply of torasemide after the trial has ended.

### Study procedures

#### Home BP monitoring.

The HBPM device (Omron M3) will be posted to the participant during screening and BP will be measured using the British & Irish Hypertension Society (BIHS) recommended method.^12^ HBPM will be measured in triplicate, morning and evening, over 5 days, during a 10-day period.^13^ The first reading from each set will be omitted and the average of the remaining recorded as HBPM. HBPM will be entered through the study website or in paper form during screening, and by completion of the HBPM diary^14^ at weeks 4 and 12.

#### Study visit BP monitoring.

Study visit BP will be measured using the method recommended by the BIHS^15^ with a validated, automated Omron BP monitor. After 5 minutes rest, 3 BP measurements will be taken, 1 minute apart with the average of the second and third recorded as visit BP.

#### Ambulatory BP monitoring.

Ambulatory BP monitoring (ABPM; Spacelabs 90217RM) over 24 hours will be measured at baseline, weeks 8 and 16 and will be used for the assessment of the study outcomes. BIHS Standard Operating Procedure, patient information leaflets and diaries will be used.^16–18^ ABPM measured for 24 hours with daytime readings every 30 minutes (0800–2159) and nighttime readings every 60 minutes (2200–0759). ABPM valid if 14 daytime measurements are obtained.^15^ With the exception of the baseline ABPM, participants must have been taking the study drug for at least 28 days prior to ABPM.

#### Genotyping.

A 2-ml saliva sample will be collected using an Isohelix GeneFiXGFX-02 kit as per as the manufacturer’s guidelines. DNA quantification will be done using NanoDrop Lite Spectrophotometer (Thermo Scientific). Genotyping for rs13333226 will be performed using TaqMan SNP Genotyping Assay ID C_31122293_10, context sequence [VIC/FAM]: GTCAAAGAGGTAGCACAGCTGTAGG [A/G]ATATTGACTCCTCTTCCCAAACAGC. DNA concentration of 5 ng/µl will be used with the TaqMan probes and TaqMan Universal Master Mix II, no UNG (Applied Biosystems) to a total reaction volume of 5 µl. This will be amplified and read using QuantStudio 12K Flex (Applied Biosystems) and the genotype read using allelic discrimination plot.

### Study samples

Blood samples will be sent to local NHS hospital laboratories. Urinary uromodulin assays will be performed, at the University of Glasgow laboratory, using a commercially available ELISA, as recommended by the manufacturer.

### Ethics approval and study registration

Study approved by the West of Scotland Research Ethics Committee 5; 16/WS/0160. The trial will be carried out in accordance with the Helsinki Declaration of 1975 (as revised in 1983). Registration at clinicaltrials.gov; trial identifier NCT03354897 and UK Clinical Research Network; GN14CE40032060. Current protocol version 2.3 (09/09/2019).

### Study objectives and statistical analysis

#### Primary outcome.

The primary outcome is the change in 24-hour ABPM SBP AUC (area under the curve) between baseline and the end of treatment.

#### Secondary outcomes.

Secondary outcomes are the change in:

- 24-hour ABPM DBP AUC between baseline and the end of treatment,
- daytime ABPM SBP and DBP AUC between baseline and the end of treatment,
- nighttime ABPM SBP and DBP AUC between baseline and the end of treatment,
- HBPM SBP and DBP AUC over the study period,
- serum electrolytes over the study period.

#### Sample size calculation.

Theoretically, the ideal study design would be the compare homozygous subjects (AA vs. GG). However, this would require screening and genotyping over 4,000 patients with uncontrolled hypertension to get adequate numbers with homozygous genotypes (as the frequency of individuals with the rarer GG genotype is (0.03) given the G allele frequency of 0.18). As the SNP shows and additive effect on BP with a population genotype frequency ratio of AA to AG/GG of 2:1, a more efficient study design to assess genotype effect on BP would be to compare AA vs. AG/GG. During the trial, the ratio of AA to AG/GG participants will be monitored along with the rate of study completion so that sufficient participants in each genotype group will be recruited to ensure robust power for the final analysis.

Sample size calculations were based on standard formulae for a normally distributed outcome, for detecting a difference in SBP between groups, assuming a SD of 8 mm Hg, a ratio of AA to AG/GG participants of 2:1, and successful follow-up of 80% of recruited participants. Under these assumptions, recruitment of 240 participants will have 90% power to detect a 4 mm Hg SBP difference and 81% power to detect a 3.5 mm Hg difference between groups. The use of baseline-adjusted regression methods will increase the power of the study. The use of 24-hour ABPM AUC as the primary outcome should also reduce within-subject variability, and hence increase power.

### Statistical analysis

Prior to database lock, study statisticians will not have access to genotypic information; statistical analyses will be specified in a detailed Statistical Analysis Plan and all statistical analysis programs will be developed and validated using dummy genotype data. Following database lock, the true genotypes will be made available for analysis. Per-protocol (PP) and intention-to-treat (ITT) analysis will be performed.

The primary analysis will use linear regression to compare mean 24-hour ABPM SBP AUC between those possessing the AA genotype and those possessing the AG/GG genotypes, with adjustment for baseline 24-hour ABPM SBP AUC. The adjusted mean difference, with a 95% confidence interval and associated *P* value, will be reported. The primary analysis will be applied to the subgroup of participants who are compliant with study procedures, including taking at least 80% of the target dose of study treatment for at least 28 days after any interruption (PP).

Secondary analyses will extend this regression model to ITT and to examine the impact of baseline patient characteristics, and to investigate the possibility of interactions with genotype. Other outcome measures will be analyzed in a similar manner. For outcomes measured at more than 1 postbaseline time point, repeated measures regression methods will also be used.

Adverse events during the trial will be summarized and listed, without formal statistical comparison. No imputation of missing data will be performed. The primary analysis will be judged at a 5% significance level. Other analyses will not be adjusted for multiple comparisons. All statistical analysis will be performed using SAS for Windows v9.3 or R for Windows v3.0 or later versions.

### Study sponsorship, monitoring, and audit

The study is sponsored by NHS Greater Glasgow and Clyde with the trial co-ordinated from the Glasgow site. Study protocol alteration will require an amendment, to be reviewed by the sponsor, with approval from research ethics committee as appropriate.

To enable regulatory authority audit, all original signed consent forms, serious adverse events, source documents, and detailed records of treatment disposition in accordance with good clinical practice principles and local regulations.

### Data collection, management, and retention

The Robertson Centre for Biostatistics (RCB) have developed the eCRF. Access is restricted via a study-specific web portal, with only authorized site-specific personnel granted permission to enter participant data. The study team will be responsible for all entries into the eCRF and will confirm (electronically) that the data are accurate and complete. Data will be validated at the point of entry into the eCRF. Any additional data discrepancies will be flagged to the principal investigator with data changes recorded to maintain a complete audit trail.

The RCB computer network is fully validated in accordance with industry and regulatory standards and incorporates controlled access security. High volume servers are firewall protected and preventative system maintenance policies are in place to ensure no loss of service. Web servers are secured by digital certificates. Data integrity is assured by strictly controlled procedures including secure data transfer procedures. Data will be retained for a minimum of 5 years.

### Dissemination

Study results will be submitted to an international conference and published in an international peer review journal (open access form). A lay summary will be available.

## DISCUSSION

The genomics revolution has resulted in over 1,400 common SNPs associated with BP along with mutations in 31 genes associated with rare monogenic forms of hypertension.^3^ Globally, about 55% of people with hypertension are receiving treatment.^19^ Tailoring of therapy has not progressed beyond considering self-reported African ancestry and serum renin levels. Strategies to reposition licensed drugs in the hypertension care pathway through genomic markers is a precision medicine strategy that can help reduce the public health burden. We propose to show that using a SNP from genome wide association study can identify patients with uncontrolled hypertension who will respond better to long acting off-patent loop-diuretics. The clinical application of this result will be a genotype-guided algorithm to determine use of loop-diuretics, and this will be relevant across the whole spectrum of hypertensive patients. As hypertension is a multifactorial disease, it is likely that the main application of our result will be earlier use of loop-diuretics in those with AA genotype. Though loop-diuretics may be less efficacious as monotherapy than other antihypertensive drug classes, they are commonly used in clinical practice for patients with difficult to control hypertension and are advocated in the European Society of Hypertension guidelines for resistant hypertension and the US guidelines as secondary agents. If the underlying pathological mechanism is an interaction between uromodulin and NKCC2 leading to increased sodium absorption in those individuals with high uromodulin excretion, then targeting NKCC2 in these individuals with loop-diuretics is a valid strategy. Such treatment is cheap, easily accessible, has an existing licence and can be clinically implemented rapidly. If this study confirms our hypothesis, a targeted strategy will improve BP control and reduce the global burden of uncontrolled hypertension.

Furthermore, the study design is novel, with genotype blinding, rather than the conventional treatment blinding, and utilizes a 2-step consent process for screening and inclusion into the main study at 3 study sites. These innovative methods place the BHF UMOD study in a unique position to inform future genotype-directed and precision medicine trials for hypertension and also nonhypertension trials.

## FUNDING

This work was supported by British Heart Foundation (CS/16/1/31878, S.P., D.J.W., T.M.Mc.D., A.Mc.).

## DISCLOSURE

The authors declared no conflict of interest.
